# Association of psychological distress and work psychosocial factors with self-reported musculoskeletal pain among secondary school teachers in Malaysia

**DOI:** 10.1371/journal.pone.0172195

**Published:** 2017-02-24

**Authors:** E. N. Zamri, F. M. Moy, V. C. W. Hoe

**Affiliations:** 1 Julius Centre University of Malaya, Department of Social & Preventive Medicine, Faculty of Medicine, University of Malaya, Kuala Lumpur, Malaysia; 2 Cluster of Lifestyle Science, Advanced of Medical & Dental Institute, Pulau Pinang, Malaysia; 3 Centre for Occupational and Environmental Health-UM, Department of Social & Preventive Medicine, Faculty of Medicine, University of Malaya, Kuala Lumpur, Malaysia; University of Tampere, FINLAND

## Abstract

**Background:**

Musculoskeletal pain is common among teachers. Work-related psychosocial factors are found to be associated with the development of musculoskeletal pain, however psychological distress may also play an important role.

**Objectives:**

To assess the prevalence of self-reported low back pain (LBP), and neck and/or shoulder pain (NSP) among secondary school teachers; and to evaluate the association of LBP and NSP with psychological distress and work-related psychosocial factors.

**Methods:**

This was a cross-sectional study conducted among teachers in the state of Penang, Malaysia. The participants were recruited via a two stage sampling method. Information on demographic, psychological distress, work-related psychosocial factors, and musculoskeletal pain (LBP and NSP) in the past 12 months was collected using a self-administered questionnaire. Poisson regression was used to estimate the prevalence ratio (PR) for the associations between psychological distress and work-related psychosocial factors with LBP and NSP.

**Results:**

The prevalence of self-reported LBP and NSP among 1482 teachers in the past 12 months was 48.0% (95% Confidence Interval (CI) 45.2%, 50.9%) and 60.1% (95% CI 57.4%, 62.9%) respectively. From the multivariate analysis, self-reported LBP was associated with teachers who reported severe to extremely severe depression (PR: 1.71, 95% CI 1.25, 2.32), severe to extremely severe anxiety (1.46, 95% CI 1.22, 1.75), high psychological job demand (1.29, 95% CI 1.06, 1.57), low skill discretion (1.28, 95% CI 1.13, 1.47) and poorer mental health (0.98, 95% CI 0.97, 0.99). Self-reported NSP was associated with mild to moderate anxiety (1.18, 95% CI 1.06, 1.33), severe to extremely severe anxiety (1.25, 95% CI 1.09, 1.43), low supervisory support (1.13, 95% CI 1.03, 1.25) and poorer mental health (0.98, 95% CI 0.97, 0.99).

**Conclusions:**

Self-reported LBP and NSP were common among secondary school teachers. Interventions targeting psychological distress and work-related psychosocial characteristics may reduce musculoskeletal pain among school teachers.

## Introduction

Musculoskeletal pain (MSP) is common among school teachers in both developed and developing countries. Previous studies found that the prevalence ranged from 20% to 95% [1–4]. The more common reported sites of MSP were neck and shoulder, low back and the upper limbs [1,4]. However, a recent systematic review suggested that research on MSP among teachers are still lacking, this is more true in Malaysia [1]. We were only able to locate three studies of MSP conducted among school teachers in Malaysia, all assessing low back pain (LBP) [5–7].

The same systematic review found that MSP among school teachers had a multifactorial origin, which included individual, physical and psychosocial factors [1]. The individual factors included female gender and increasing age, which was found to be positively associated with MSP. Meanwhile, poor postures, inappropriate workstations, lifting and carrying heavy objects were the common work-related physical factors. The work-related psychosocial factors identified were high psychological job demands, low job control and low social support.

Other than the above mentioned factors, psychological distress is another factor that needs to be considered. The concept of psychological distress is a broad label given to a variety of states and responses related to depression and anxiety. Previous research indicated that there was a high prevalence of psychological distress among school teachers, however the evidence on the relationship between psychological distress and MSP is still lacking [5,8,9].

Hence, we aimed to determine the prevalence of self-reported LBP and NSP and to explore the association between psychological distress and work-related psychosocial factors with LBP and NSP among school teachers.

## Material and methods

### Study design

This was a cross-sectional study conducted from January to March 2014. Data was collected using self-administered questionnaires and anthropometric measures such as weight and height were measured following standard protocols. This study is the baseline component of the prospective cohort study on Clustering of Lifestyle risk factors and Understanding its association with Stress on health and wellbeing among school Teachers in Malaysia (CLUSTer)[10]. CLUSTer was conducted among school teachers in Malaysia, intended to explore the clustering of lifestyle risk factors and stress, and its association with major chronic medical conditions such as obesity, hypertension, impaired glucose tolerance, diabetes mellitus, coronary heart diseases, kidney failure and cancers.

### Study population

The study population consists of teachers from all public secondary schools in the state of Penang, Malaysia. The state of Penang is made up of five districts with a total of 101 public secondary schools. A two-stage sampling method was employed. First,70% of the public schools from each district was randomly selected and in the second stage, all the eligible teachers in the schools which have agreed to participate were invited for the study.

### Recruitment process

In the first stage, after the schools were selected; an invitation letter, information sheets describing the study, the permission letter from the Ministry of Education Malaysia and Penang Education Department were sent to the heads of the selected schools. Out of the 71 selected secondary schools, 57 secondary schools agreed to participate. In the second stage, universal sampling was employed. All tenured teachers in the participating schools were eligible, teachers employed on contract basis and those who were pregnant were excluded. The participation of the schools and teachers were entirely voluntary. Ethics clearance was obtained from the Medical Ethics Committee of the University Malaya Medical Centre (Reference Number: MEC 950.1). Written informed consent was obtained from all participants prior to data collection.

### Study instruments

#### Measurement of Musculoskeletal Pain (MSP)

The symptoms on MSP were assessed using the modified Nordic Musculoskeletal Questionnaire (NMQ). The original NMQ consists of two sections; the first section is a general questionnaire of 40 forced-choice items identifying areas of the body causing musculoskeletal problems, and the second section consists of 25 forced-choice additional questions relating to the neck, shoulders and lower back which detail issues such as any accidents affecting each area, functional impact at home and work (change of job or duties), duration of the problem, assessment by a health professional and musculoskeletal problems in the last 7 days [11]. The modified NMQ has six questions enquiring if participants had experienced pain in the lower back, neck and/or shoulder (depicted in diagrams) in the preceding one month and 12 months with binary response (yes/no). However, in this study, we only reported the 12-month prevalence of LBP and NSP. The NMQ appears as the accepted method used commonly to measure the prevalence of MSP.

#### Measurement of psychological factors (psychological distress and mental health)

Psychological distress such as depression, anxiety and stress were assessed with the culturally adapted and validated 21-item Depression Anxiety Stress Scale (DASS21) in the Malay language [12]. DASS21 was proven to be valid in both clinical and community settings in English-speaking countries [13–16]. The internal consistency of DASS21 in Malay language had Cronbach’s alpha values of 0.84, 0.74 and 0.79 for depression, anxiety and stress scales respectively [12]. The responses for each item ranged from 0 (did not apply to me at all) to 3 (applied to me very much and most of the time). The total score for each subscale was calculated and the severity rating was classified as normal, mild to moderate, severe to extremely severe.

Self-perceived mental health was measured using the Mental Component Summary Scale (SF-12 MCS) of the 12-item Short Form Health Survey (SF12v2) [17]. The instrument has good internal consistency (Cronbach’s alpha = 0.70) for the Malay version of SF-12 MCS [18]. The scoring of SF-12 MCS was calculated using the Quality Metric Health Outcomes Scoring Software. Higher score indicating better mental health.

#### Measurement of work-related psychosocial factors

Work-related psychosocial factor was assessed using the validated Malay version of the Job Content Questionnaire (JCQ). It demonstrated poor to good internal consistency with Cronbach’s alpha values ranged between 0.50 and 0.84 [19]. JCQ is a 22-item questionnaire with responses for each item ranging from 1 (strongly disagree) to 4 (strongly agree). There are five subscales measured in JCQ, namely decision authority (three items), psychological job demand (five items), skill discretion (six items), co-worker support (four items) and supervisor support (four items). The scores for each of the scale were calculated using the recommended formula [20]. Then, the sum of scores for each scale was dichotomised based on the median score. For example, a score above the sample median on psychological job demands was considered as ‘high’ meanwhile below the sample median considered as ‘low’.

#### Measurement of socio-demographic characteristics, co-morbidities and health related behaviours (smoking status & physical activity)

Socio-demographic characteristics such as age, gender and marital status were assessed using the self-administered questionnaire. Information on medical conditions diagnosed by physicians such as diabetes mellitus, hypertension, cardiovascular disease and hypercholesterolemia were self-reported. The participants’ current smoking status was also enquired.

Physical activity level for the preceding seven days was assessed with the Malay version of the 7-item International Physical Activity Questionnaire (IPAQ). The total daily activities were computed based on IPAQ scoring guidelines and was categorised as low (<600 MET-min/week), moderate (600–1499 MET-min/week) and vigorous (≥1500 MET-min/week) activity [21]. The instrument had good reliability with intraclass correlation coefficients (ICC) ranging from 0.75 to 0.93 [22].

#### Measurement of anthropometric parameters

The participants’ weight was measured by trained field research assistants using the Tanita TBF-310 Body Composition Analyser, with light clothing but shoes and socks removed. Height was measured with a portable stadiometre (SECA 217, Hamburg, Germany) without shoes [10]. Body Mass Index (BMI) was calculated with the formula of weight (kg)/ height (metre)^2^ and was classified as underweight (BMI <18.5 kg/m^2^), normal weight (BMI 18.5–24.9 kg/m^2^), overweight (BMI 25.0–29.9 kg/m^2^) and obese (BMI ≥30.0 kg/m^2^) [23].

### Statistical analysis

Since complex sampling was used, sampling weight was applied to correct for unequal selection probabilities and non-response to produce unbiased estimates. Information on total schools, total schools participated, total teachers in all schools and total teachers participated were collected in order to calculate the sampling weights.

Frequency and percentage were presented for categorical variables while mean and standard deviation for normally distributed continuous variables. All statistical tests were two-sided with the significant level pre-set at p <0.05. Confidence intervals (CIs) were estimated at the 95% level. The statistical analyses were performed using Poisson regression with robust estimates of variance to identify association between individual and work-related factors; with LBP and NSP.As the prevalence of LBP and NSP was high (>10%), prevalence ratio (PRs) was used instead of odds ratio. Odds ratio tends to over-estimate the strength of association when the outcome is common.

Variables that were significant in the univariate analysis were included in the multivariate analysis. Interaction term between job demand and job resources was tested in the univariate analysis as the central hypothesis of job demands-resources (JD-R) model proposed interaction between high demands and low job resources might affect employee’s health and well-being [24]. If the result showed non-significance in the preliminary full model, the interaction term would be dropped from the final model. The analysis was performed using the Stata Software (Stata Corp., LP, College Station, TX), version 11.0.

## Results

**Fig 1:**
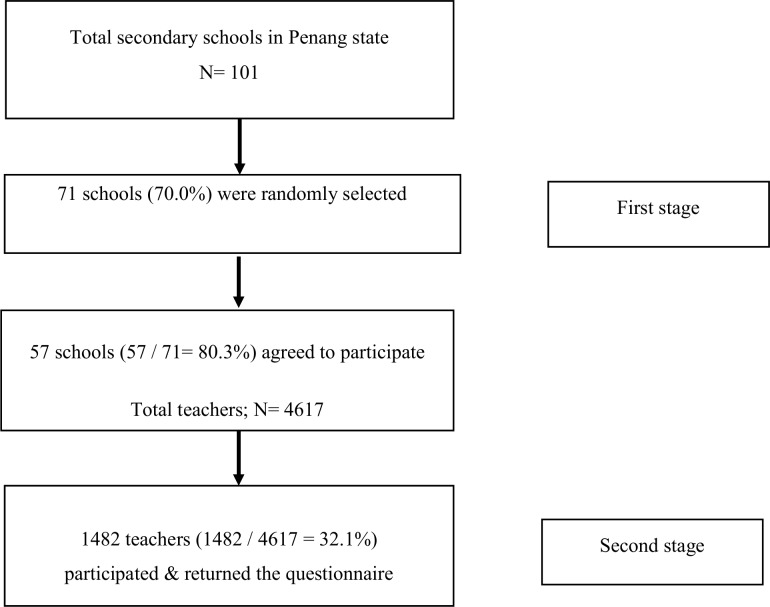
Flow chart of recruitment process.

The response rates for schools and teachers were 80.3% and 32.1% respectively (Fig 1). The 12-month prevalence of self-reported low back pain (LBP) and neck and/or shoulder pain (NSP) were 48.0% (95% CI: 45.2, 50.9) and 60.1% (95% CI: 57.4, 62.9) respectively. The mean (standard deviation) age of participants was 41.2 (8.74) years old. Majority of them were females (81.7%) and non-smoker (98.2%). The ethnic distribution reflected the country’s distribution in which Malays (69.2%) were the largest group, followed by Chinese (21.9%) and others (8.9%) (Table 1).

**Table 1:** Distribution of participants’ characteristics by demographic, individual and work related factors.

Variable	Total participants; n (%)*	Self-reported LBP (n = 1482); n (%)*	p-value	Self-reported NSP (n = 1482); n (%)*	p- value
**Prevalence (95%CI)**		48.0 (45.2–50.9)		60.1 (57.4–62.9)	
**Age Mean (±SD)** ^§^	41.2 (8.74)	40.06 (8.61)	<0.001	41.02 (8.55)	0.440
**Age (years)**^+^			0.002		0.543
20–29	145 (11.2)	80 (6.4)		84 (6.5)	
30–39	467 (32.2)	240 (16.8)		291 (20.2)	
40–49	573 (36.7)	268 (17.1)		336 (22.1)	
≥ 50	297 (19.9)	121 (7.8)		171 (11.4)	
**Gender**^+^			0.982		0.608
Male	295 (18.3)	145 (8.8)		166 (10.7)	
Female	1187 (81.7)	564 (39.3)		716 (49.4)	
**Ethnicity**^+^			0.419		0.241
Malay	1056 (69.2)	498 (33.1)		610 (40.5)	
Chinese	293 (21.9)	138 (10.2)		185 (14.0)	
Others	133 (8.9)	73 (4.8)		87 (5.7)	
**Level of physical activity (MET-min/week)**^+^			0.568		0.389
Low (<600)	353 (29.1)	160 (13.3)		202 (16.8)	
Moderate (600–1499)	247 (19.5)	120 (9.8)		139 (11.3)	
High (>1500)	654 (51.4)	315 (25.1)		397 (31.9)	
**Body mass index (kg/m**^**2**^**)**^+^			0.984		0.781
Low (<18.5)	19 (1.7)	10 (0.9)		11 (1.1)	
Normal (18.5–24.9)	420 (29.5)	197 (14.2)		260 (18.4)	
Overweight (25.0–29.9)	600 (39.7)	288 (18.8)		357 (23.7)	
Obesity (>30.0)	443 (29.1)	214 (14.2)		254 (17.0)	
**Current smoker**^+^	28 (1.8)	17 (1.1)	0.098	20 (1.4)	0.052
**Co-morbidities**^+^					
Cardiovascular disease	9 (0.5)	3 (0.2)	0.541	7 (0.4)	0.237
Diabetes mellitus	58 (3.5)	35 (2.1)	0.060	35 (2.2)	0.814
Hypercholesterolemia	205 (13.9)	94 (6.2)	0.352	128 (8.5)	0.802
Hypertension	103 (6.6)	43 (2.7)	0.142	70 (4.6)	0.040
**Depression**^+^			<0.001		<0.001
Normal	1081 (72.7)	465 (30.6)		599 (40.1)	
Non-severe (mild to moderate	353 (24.4)	211 (14.7)		242 (17.1)	
Severe (Severe to extremely severe)	35 (2.9)	27 (2.4)		31 (2.6)	
**Anxiety**^+^			<0.001		<0.001
Normal	971 (66.2)	401 (27.1)		517 (35.8)	
Non-severe (mild to moderate)	305 (21.1)	164 (11.6)		205 (14.2)	
Severe (severe to extremely severe)	193 (12.7)	138 (9.2)		151 (10.1)	
**Stress**^+^			<0.001		<0.001
Normal	1194 (80.4)	526 (34.9)		675 (45.2)	
Non-severe (mild to moderate)	233 (16.6)	149 (10.9)		163 (12.2)	
Severe (severe to extremely severe)	42(3.0)	27 (2.0)		35 (2.5)	
**Working characteristics**					
**Teaching years**^+^			0.013		0.188
<15	694(48.9)	346 (25.3)		424 (30.4)	
≥15	781 (51.1)	358 (22.7)		455 (29.9)	
**Teaching hours (per day)**^+^			0.087		0.138
<4	589 (40.8)	267 (18.5)		337 (23.6)	
≥4	862 (59.2)	428 (29.8)		529 (36.8)	
**Administrative working hour (per day)**^+^			0.547		0.880
<5	665 (51.2)	316 (24.6)		398 (31.1)	
≥5	656 (48.8)	322 (24.5)		389 (29.5)	
**Work Psychosocial factors**					
**Decision authority**^+^			0.886		0.669
Low decision	369 (25.9)	180 (12.4)		226 (15.9)	
High decision	1095 (74.1)	521 (35.8)		644 (44.3)	
**Skill discretion**^+^			0.007		0.559
Low skill	319 (22.3)	171 (12.4)		191 (13.8)	
High skill	1110 (77.7)	514 (35.8)		661 (46.5)	
**Psychological job demand**^+^			0.007		0.059
High job demand	1257 (85.1)	618 (42.3)		762 (52.1)	
Low job demand	211 (14.9)	85 (5.8)		111 (7.9)	
**Supervisor Support**^+^			0.157		0.001
Low support	438 (30.2)	221 (15.6)		284 (20.4)	
High support	1028 (69.8)	482 (32.7)		589 (39.9)	
**Co-worker support**^+^			0.358		0.390
Low support	195 (13.1)	103 (6.7)		126 (8.3)	
High support	1273 (86.9)	600 (41.4)		747 (51.8)	
**Self-perceived mental health**					
Mental component summary score (SF-12 MCS), Mean (±SD) ^§^	48.26 (8.2)	46.92 (8.00)	<0.001	47.21 (8.1)	<0.001

NSP–Neck and/or Shoulder Pain; LBP–Low back pain.

SD–Standard deviation.

*n–unweighted count; %–weighted percentage.

CI–Confidence Interval.

^+^ analysed using Chi square test.

^§^ analysed using Independent T-test.

There were slightly higher proportions from those who had ≥ 15 years’ experience in teaching, taught ≥ 4 hours/day, spent <5 hours/day in administrative work. More than half reported that they were involved in high physical activity, however majority were still overweight and obese. The proportions on comorbidities were low except hypercholesterolemia which was slightly more than ten percent.

Majority of the teachers perceived that they had no issues in depression and stress. However, slightly less than half of them reported to have problem with anxiety. Majority of the teachers reported to have high decision authority, high skill discretion, high psychological job demand, high supervisor and co-worker support.

Those aged 40 years and above had significantly lower odds of self-reported LBP, meanwhile diabetes mellitus (DM) had higher odds of self-reported LBP (Table 2). There was no significant association between socio-demographic factors and self-reported NSP. Hypertension and current smokers were found to have higher odds of self-reported NSP. Psychological distress namely symptoms of depression, anxiety and stress (either non severe or severe) demonstrated significant association with self–reported LBP and NSP. The SF-12 MCS was inversely associated with LBP and NSP. Teaching less than 15 years, low skill discretion and high job demand were associated with self-reported LBP, while low supervisor support was significantly associated with self-reported NSP. There was a significant interaction effect between high job demand and low job resources (low skill discretion) with LBP.

**Table 2:** Univariate analysis using associated factors with self-reported Low Back Pain (LBP) and Neck and/or Shoulder Pain (NSP) in the past 12 months.

	Prevalence ratio (95% CI)	
Variables	Low back pain	Neck and/or Shoulder pain
**Age (years)**		
20–29	1.00	1.00
30–39	0.90 (0.74–1.10)	1.02 (0.83–1.25)
40–49	0.81 (0.66–0.98)*	1.10 (0.96–1.26)
≥50	0.67 (0.53–0.84)*	1.06 (0.93–1.21)
**Gender**		
Male	1.00	1.00
Female	0.96 (0.82–1.11)	1.02 (0.91–1.15)
**Ethnicity**		
Malay	1.00	1.00
Chinese	1.08 (0.91–1.29)	1.11 (0.98–1.26)
Others	1.21 (1.00–1.45)	1.10 (0.95–1.29)
**Level of physical activity (MET-min/week)**		
Low (<600)	0.93 (0.80–1.09)	0.92 (0.82–1.04)
Moderate (600–1499)	1.03 (0.88–1.22)	0.93 (0.81–1.06)
High (>1500)	1.00	1.00
**Body Mass Index (kg/m**^**2**^**)**		
Low (<18.5)	1.00	1.00
Normal (18.5–24.9)	0.98 (0.59–1.64)	1.01 (0.68–1.49)
Overweight (25.0–29.9)	0.98 (0.59–1.62)	0.97 (0.66–1.43)
Obesity (>30.0)	1.01 (0.60–1.67)	0.95 (0.64–1.40)
**Current smoker**	1.35 (0.97–1.87)	1.34 (1.08–1.68)*
**Morbidities**		
Cardiovascular disease	0.86 (0.35–2.09)	1.35 (0.96–1.90)
Diabetes mellitus	1.39 (1.11–1.76)*	1.06 (0.85–1.32)
Hypercholesterolemia	0.99 (0.83–1.19)	1.04 (0.91–1.19)
Hypertension	0.89 (0.68–1.16)	1.23 (1.07–1.42)*
**Depression**		
Normal	1.00	1.00
Mild to moderate	1.40 (1.24–1.58)*	1.27 (1.15–1.39)*
Severe to extremely severe	1.98 (1.67–2.36)*	1.66 (1.48–1.85)*
**Anxiety**		
Normal	1.00	1.00
Mild to moderate	1.33 (1.15–1.53)*	1.25 (1.12–1.39)*
Severe to extremely severe	1.73 (1.52–1.97)*	1.47 (1.32–1.63)*
**Stress**		
Normal	1.00	1.00
Mild to moderate	1.48 (1.31–1.68)*	1.25 (1.12–1.39)*
Severe to extremely severe	1.49 (1.11–1.99)*	1.47 (1.32–1.63)*
**Self-perceived mental health**		
Mental component summary score (SF-12 MCS)	0.98 (0.97–0.99)*	0.98 (0.97–0.99)*
**Working characteristics**		
Teaching years (≤15 years)	1.16 (1.03–1.31)	0.93 (0.82–1.07)
Teaching hours (per day)	1.08 (0.95–1.22)	1.07 (0.97–1.18)
Administrative working hour (per day)	1.03 (0.91–1.17)	0.99 (0.89–1.09)
**Work Psychosocial factors**		
**Skill discretion**		
Low—skill	1.23 (1.08–1.39)*	1.04 (0.93–1.15)
High—skill	1.00	1.00
**Psychological job demand**		
High job demand	1.23 (1.02–1.49)*	1.13 (0.98–1.31)
Low job demand	1.00	1.00
**Supervisor Support**		
Low support	1.09 (0.97–1.24)	1.18 (1.07–1.29)*
High support	1.00	1.00
**Co-worker support**		
Low support	1.08 (0.91–1.29)	0.94 (0.82–1.08)
High support	1.00	1.00
**Decision authority**		
Low authority	1.00 (0.87–1.15)	1.03 (0.92–1.15)
High authority	1.00	1.00
**High Job demand × Low skill discretion**	1.44 (1.09–1.89)*	NA

*p<0.05.

LBP was significantly associated with diabetes mellitus, self-reported severe depression and anxiety, work-related psychosocial factors such as low skill discretion and high psychological job demand in the final model; after adjusted for gender, age, current smoking status, diabetes mellitus, teaching hours and teaching years (Table 3). In addition, age and the SF-12 MCS were inversely associated with LBP. The interaction term of job demand and job resources (low skill discretion) was removed from the final model of LBP as it was not statistically significant in the preliminary full model.

**Table 3:** Multivariate analysis of self-reported Low Back Pain (LBP) and Neck and/or Shoulder Pain (NSP) in the past 12 months.

	Prevalence Ratio (95%CI)	
	Low back pain^1^	Neck and/or shoulder pain ^2^
**Age (years)**		
20–29	1.0	1.0
30–39	0.97 (0.80–1.17)	1.09 (0.91–1.29)
40–49	0.76 (0.59–0.98)*	1.10 (0.89–1.35)
≥50	0.66 (0.49–0.89)*	1.07 (0.84–1.36)
**Female**	0.93 (0.80–1.09)	1.07 (0.94–1.22)
**Smoker**	1.16 (0.84–1.59)	1.39 (1.11–1.74)*
**Teaching hours (>4hours/day)**	1.16 (0.93–1.20)	1.02 (0.92–1.12)
**Teaching years (≤15 years)**	0.83 (0.68–1.03)	1.02 (0.88–1.17)
**Diabetes mellitus**	1.40 (1.13–1.74)*	NA
**Hypertension**	NA	1.16 (1.01–1.35)*
**Depression**		
Normal	1.0	1.0
Mild to moderate	1.15 (0.98–1.36)	1.12 (0.99–1.27)
Severe to extremely severe	1.71 (1.25–2.32)*	1.37 (1.09–1.71) *
**Anxiety**		
Normal	1.0	1.0
Mild to moderate	1.22 (1.05–1.42)*	1.18 (1.06–1.33)*
Severe to extremely severe	1.46 (1.22–1.75)*	1.25 (1.09–1.43)*
**Stress**		
Normal	1.0	1.0
Mild to moderate	1.03 (0.86–1.25)	0.98 (0.86–1.13)
Severe to extremely severe	0.72 (0.49–1.04)	0.94 (0.76–1.19)
**High psychological job demand**	1.29 (1.06–1.57)*	NA
**Low skill discretion**	1.28 (1.13–1.47)*	NA
**Low supervisor support**	NA	1.13 (1.03–1.25)*
**Mental Component summary score (SF-12 MCS)**	0.99 (0.97–0.99)*	0.98 (0.97–0.99)*

^1^Adjusted for age, gender, diabetes mellitus, current smoking status, teaching years, and teaching hours.

^2^ Adjusted for age, gender, hypertension, current smoking status, teaching years, and teaching hours.

NA- not applicable.

*p<0.05.

In the final model of NSP (Table 3) after adjusted for gender, age, current smoking status, hypertension, teaching hours and teaching years, NSP was significantly associated with smoking status, anxiety and depression and low supervisor support. Similar with LBP, SF-12 MCS was inversely associated with NSP.

## Discussion

Our results showed that the 12-month prevalence of self-reported LBP is comparable to other Malaysian studies on LBP among primary school teachers, ranging between 40.4% and 72.9% [5–7]. The prevalence of LBP was also comparable with most studies conducted in Asia, which ranged between 20% and 53% [25–28], except for a study from Japan that reported a low prevalence of 20.6% [27]. This may be due to cultural influences as another study too found a four-fold difference in the prevalence of LBP among the Japanese nurses (11.3%) compared to nurses in Costa Rica (37.7%) and Nicaragua (42.6%) [29].

The 12-month prevalence of NSP (60.1%) was higher than LBP (48.0%) among our participants. There were only few studies that examined the prevalence of NSP among teachers. Most studies measured neck and shoulder pain separately. Two studies from Hong Kong found between 64.4% and 66.7% of secondary school teachers reported to suffer from neck pain [9,30]. Meanwhile, a study in Estonia found the prevalence of neck and shoulder pain among school teachers was 33.3% and 7.8% respectively [31]. Only one study reported both neck and/or shoulder pain together (NSP) with a prevalence of 57.9% among teachers from China [25], comparable with our results.

Psychological factors have been found to play an important role in the development of back and neck pain [32]. Previous studies found that psychological distress was associated with MSP among various groups of working population [33–36] including school teachers [5,8,9]. In our study, there was an increasing trend in the proportions of both LBP and NSP with increase in the score of self-reported depression and anxiety. Depression and anxiety are considered as an internalizing type of psychological distress [37]. Some researchers suggested the association of psychological distress and MSP might be due to the influence of work-related psychosocial factors [38–40]. However, we found no interaction between psychological distress and work-related psychosocial factors in the association of MSP (data not shown). Therefore, we postulate that psychological distress and work-related psychosocial factors were independently associated with MSP among our study participants. Besides that, non-work factor might have an influence in the association between psychological distress and MSP. The non-work factors include satisfaction with one’s social life, social support from spouse and relatives, stressful life events and past experience related to pain.

The SF-12 MCS score was used to assess health-related quality of life in terms of mental health. Our findings indicated that poor mental health was associated with self-reported LBP and NSP. Previous studies showed that mental health was a strong predictor of development and persistence in pain [41] and individuals with disability due to LBP was a risk factor for poor mental health [42]. However, we could not determine whether mental health is a cause or consequence of MSP since our cross section design could not establish causality. Therefore, longitudinal study design should be carried out to understand the contribution of these psychological influences if preventive measures are to be optimized.

Along with psychological factors, we have assessed the role of workplace psychosocial factors on MSP. We found high job demand was only associated with LBP but not NSP. Our finding is not consistent with a previous review [43] which found that high job demands was the most consistent findings associated with back pain and neck and/or shoulder pain. The association between high job demand and LBP might be due to the nature of the work of school teachers which required physical demand to complete their task. According to Bugajska et al. [44] when the physical work load reduced, there would be a reduce impact between job demand and onset of upper limb symptoms. However, we did not consider work-related physical factors in our study.

We found younger teachers were more susceptible to LBP compared to the older age groups, as reported elsewhere [25]. Younger teachers might face greater work demand as they were given more tasks at the beginning of their career [45]. Meanwhile, Chiu & Lam [9] suggested that young teachers might not be adapting well to the new working environment, and this eventually increased their physical and psychological stress that might affect their musculoskeletal conditions. However, we did not find any significant association between age and NSP. NSP may be less affected by age, further investigation should be conducted to ascertain this in more details.

It is important to note that psychological distress and work-related psychosocial factors were independently associated with MSP. Although our results demonstrated that different pain sites had different associated factors, there was a consistency where psychological distress and work-related psychosocial factors might play an important role on LBP and NSP among school teachers. Therefore, the availability of educational psychologists should be provided and made known to teachers with symptoms of depression and anxiety. Workshops or seminars on stress management should be conducted routinely as a preventive measure. Physical activity program in workplace should be conducted as it may reduce MSP and improve mental health and quality of life.

### Strengths & limitations

There are some limitations that warrant discussion. Causality cannot be established as our study was of cross-sectional design. Selection bias might occur where teachers who volunteered may have different characteristics compared to the non-respondents. However, we did not collect detailed information on the non-respondents. There is a possibility of recall bias, since the instruments used were self-reported and subjective. However, most of the instruments used were established and validated both internationally and locally, except JCQ in the Malay version had relatively poor to good internal consistency across its subscales [19]. This may be due to the validation was conducted among teachers in one state only. Future research should be conducted to validate the JCQ in the Malay version among our population in more states within our country. Common method variance may occur as the same source (questionnaire survey) was used to assess MSP, psychological and psychosocial factors from the participants. However, different scales were used for NMQ, DASS21, SF-12, and JCQ which may reduce the possibility of common method variance.

On the other hand, our study may be the first in our country assessing the prevalence of LBP and NSP concurrently and its association between psychological distress and work psychosocial factors among school teachers. The two-stage sampling method used ensured representation of all secondary school teachers in the state. The large sample size provided adequate power for the study. The use of PR instead of OR ensured that we did not over-estimate the strength of association between variables [46].

## Conclusion

Our findings indicated that self-reported LBP and NSP were common among secondary school teachers. Psychological distress and work related psychosocial factors were both associated with self-reported LBP and NSP. Different sites of MSP had different sets of associated factors. Future research with a longitudinal design should be carried out to establish the causal effect of psychological and work-related psychosocial factors in the development of MSP among teachers. Furthermore, research on the effectiveness of psychological intervention to reduce MSP in teachers should be considered.

## References

[1] Erick PN, Smith DR (2011) A systematic review of musculoskeletal disorders among school teachers. BMC Musculoskelet Disord 12: 260 10.1186/1471-2474-12-260 22087739 PMC3250950

[2] Rottermund J, Knapik A, Saulicz E, Myśliwiec A, Saulicz M, Rygiel KA, et al (2015) Back and neck pain among school teachers in Poland and its correlations with physical activity. Med Pr 66: 771–778. 10.13075/mp.5893.00121 26674164

[3] Erick PN, Smith DR (2014) Low back pain among school teachers in Botswana, prevalence and risk factors. BMC Musculoskelet Disord 15: 359 10.1186/1471-2474-15-359 25358427 PMC4230345

[4] Darwish MA, Al-Zuhair SZ (2013) Musculoskeletal Pain Disorders among Secondary School Saudi Female Teachers. Pain Res Treat 2013: 7. 10.1155/2013/878570 PMC3736412 23970968

[5] Nurul Izzah AS, Abdullah H, Moin S, Shamsul Bahri MT, Hashim Z (2010) Prevalence of Low Back Pain and its Risk Factors among School Teachers. American Journal of Applied Sciences 7: 634–639.

[6] Nur Farahwahida MA, Irniza R, Suhainizam MS, Emilia ZA (2016) Work Task and Job Satisfaction Predicting Low Back Pain among Secondary School Teachers in Putrajaya. Iranian Journal of Public Health 45: 85–92.

[7] Rajan B, Martin EC, Thenmozhi(2016) Prevalence of low back pain and its risk factors among secondary school teachers at Bentong, Pahang. International Journal of Physical Education, Sports and Health 3: 35–40.

[8] Korkmaz NC, Cavlak U, Telci EA (2011) Musculoskeletal pain, associated risk factors and coping strategies in school teachers. Scientific Research and Essays 6: 649–657.

[9] Chiu TT, Lam PK (2007) The prevalence of and risk factors for neck pain and upper limb pain among secondary school teachers in Hong Kong. J Occup Rehabil 17: 19–32. 10.1007/s10926-006-9046-z 16933144

[10] Moy FM, Hoe VC, Hairi NN, Buckley B, Wark PA, Koh D, et al (2014) Cohort study on clustering of lifestyle risk factors and understanding its association with stress on health and wellbeing among school teachers in Malaysia (CLUSTer)—a study protocol. BMC Public Health 14: 611 10.1186/1471-2458-14-611 24938383 PMC4081548

[11] Crawford JO (2007) The Nordic Musculoskeletal Questionnaire. Occupational Medicine 57: 300–301.

[12] Ramli M, Mohd Ariff F, Zain Z (2007) Translation, validation and psychometric properties of Bahasa Malaysia version of the Depression Anxiety and Stress Scales (DASS). ASEAN Journal of Psychiatry 8: 82–89.

[13] Ng F, Trauer T, Dodd S, Callaly T, Campbell S, Berk M (2007) The validity of the 21‐item version of the Depression Anxiety Stress Scales as a routine clinical outcome measure. Acta Neuropsychiatrica 19: 304–310. 10.1111/j.1601-5215.2007.00217.x 26952943

[14] Brown TA, Chorpita BF, Korotitsch W, Barlow DH (1997) Psychometric properties of the Depression Anxiety Stress Scales (DASS) in clinical samples. Behaviour research and therapy 35: 79–89. 9009048 10.1016/s0005-7967(96)00068-x

[15] Antony MM, Bieling PJ, Cox BJ, Enns MW, Swinson RP (1998) Psychometric properties of the 42-item and 21-item versions of the Depression Anxiety Stress Scales in clinical groups and a community sample. Psychological Assessment 10: 176–181.

[16] Henry JD, Crawford JR (2005) The short‐form version of the Depression Anxiety Stress Scales (DASS‐21): Construct validity and normative data in a large non‐clinical sample. British journal of clinical psychology 44: 227–239. 10.1348/014466505X29657 16004657

[17] Ware JE Jr, Kosinski M, Keller SD (1996) A 12-Item Short-Form Health Survey: construction of scales and preliminary tests of reliability and validity. Medical care 34: 220–233. 8628042 10.1097/00005650-199603000-00003

[18] Norhayati MN, Aniza AA (2014) Validity and Reliability of The Malay Version Of 12-Item Short Form Health Survey among Postpartum Mothers. Malaysian Journal of Public Health Medicne 14: 56–66.

[19] Hadi AA, Naing NN, Daud A, Nordin R (2006) Reliability and construct validity of the Malay version of the Job Content Questionnaire (JCQ) among secondary school teachers in Kota Bharu, Kelantan, Malaysia. Southeast Asian J Trop Med Public Health 37: 1254–1259. 17333785

[20] Karasek RA (1985) Job content questionnaire and user’s guide. Lowell: University of Massachusetts, Department of Work Environment.

[21] IPAQ CR (2005) Guidelines for Data Processing and Analysis of the International Physical Activity Questionnaire (IPAQ)–Short and Long Forms

[22] Papathanasiou G, Georgoudis G, Papandreou M, Spyropoulos P, Georgakopoulos D, Kalfakakou V, et al (2009) Reliability measures of the short International Physical Activity Questionnaire (IPAQ) in Greek young adults. Hellenic J Cardiol 50: 283–294. 19622498

[23] WHO (2000) Obesity: preventing and managing the global epidemic. World Health Organization. 11234459

[24] Schaufeli WB, Taris TW (2014) A critical review of the Job Demands-Resources Model: Implications for improving work and health. Bridging occupational, organizational and public health: Springer pp. 43–68.

[25] Yue P, Liu F, Li L (2012) Neck/shoulder pain and low back pain among school teachers in China, prevalence and risk factors. BMC Public Health 12: 789 10.1186/1471-2458-12-789 22978655 PMC3524038

[26] Jin K, Sorock GS, Courtney TK (2004) Prevalence of low back pain in three occupational groups in Shanghai, People's Republic of China. J Safety Res 35: 23–28. 10.1016/j.jsr.2003.11.002 14992843

[27] Tsuboi H, Takeuchi K, Watanabe M, Hori R, Kobayashi F (2002) Psychosocial factors related to low back pain among school personnel in Nagoya, Japan. Ind Health 40: 266–271. 12141375 10.2486/indhealth.40.266

[28] Atlas A, Bondoc R, Garrovillas R, Lo R, Recinto J, Yu K, et al (2007) Prevalence of low back pain among public high school teachers in the City of Manila. Philippine Journal of Allied Health Sciences 2: 34–40.

[29] Coggon D, Ntani G, Palmer KT, Felli VE, Harari R, Barrero LH, et al (2013) Disabling musculoskeletal pain in working populations: Is it the job, the person, or the culture? PAIN 154: 856–863. 10.1016/j.pain.2013.02.008 23688828 PMC3675684

[30] Chiu T, Lau K, Ho C, Ma M, Yeung T, Cheung P (2006) A study on the prevalence of and risk factors for neck pain in secondary school teachers. Public Health 120: 563–565. 10.1016/j.puhe.2006.01.007 16684548

[31] Pihl E, Matsin T, Jurimae T (2002) Physical activity, musculoskeletal disorders and cardiovascular risk factors in male physical education teachers. J Sports Med Phys Fitness 42: 466–471. 12391442

[32] Linton SJ (2000) A review of psychological risk factors in back and neck pain. Spine (Phila Pa 1976) 25: 1148–1156. 10788861 10.1097/00007632-200005010-00017

[33] Leino P, Magni G (1993) Depressive and distress symptoms as predictors of low back pain, neck-shoulder pain, and other musculoskeletal morbidity: a 10-year follow-up of metal industry employees. Pain 53: 89–94. 8316395 10.1016/0304-3959(93)90060-3

[34] Nahit ES, Hunt IM, Lunt M, Dunn G, Silman AJ, Macfarlane GJ (2003) Effects of psychosocial and individual psychological factors on the onset of musculoskeletal pain: common and site-specific effects. Ann Rheum Dis 62: 755–760. 10.1136/ard.62.8.755 12860731 PMC1754627

[35] Carlisle KN, Parker AW (2014) Psychological distress and pain reporting in Australian coal miners. Saf Health Work 5: 203–209. 10.1016/j.shaw.2014.07.005 25516813 PMC4266779

[36] Feyer A, Herbison P, Williamson A, de Silva I, Mandryk J, Hendrie L, et al (2000) The role of physical and psychological factors in occupational low back pain: a prospective cohort study. Occupational and Environmental Medicine 57: 116–120. 10.1136/oem.57.2.116 10711279 PMC1739913

[37] Moore SA, Dowdy E, Furlong MJ (2016) Using the Depression, Anxiety, Stress Scales–21 With U.S. Adolescents: An Alternate Models Analysis. Journal of Psychoeducational Assessment: 1–18.

[38] Lavoie-Tremblay M, Bonin JP, Lesage AD, Bonneville-Roussy A, Lavigne GL, Laroche D (2010) Contribution of the psychosocial work environment to psychological distress among health care professionals before and during a major organizational change. Health Care Manag (Frederick) 29: 293–304. 21045581 10.1097/HCM.0b013e3181fa022e

[39] Hoogendoorn WE, Bongers PM, de Vet HC, Houtman IL, Ariëns GA, van Mechelen W, et al (2001) Psychosocial work characteristics and psychological strain in relation to low-back pain. Scand J Work Environ Health 27: 258–267. 11560340 10.5271/sjweh.613

[40] Bongers PM, de Winter CR, Kompier MA, Hildebrandt VH (1993) Psychosocial factors at work and musculoskeletal disease. Scandinavian journal of work, environment & health: 297–312. 10.5271/sjweh.1470 8296178

[41] Palmer KT, Reading I, Linaker C, Calnan M, Coggon D (2008) Population-based cohort study of incident and persistent arm pain: role of mental health, self-rated health and health beliefs. Pain 136: 30–37. 10.1016/j.pain.2007.06.011 17689865 PMC3284249

[42] Rodriguez-Romero B, Pita-Fernandez S, Pertega-Diaz S (2015) Impact of musculoskeletal pain on health-related quality of life among fishing sector workers. Clin Rheumatol 34: 1131–1139. 10.1007/s10067-014-2550-1 24647978

[43] Macfarlane GJ, Pallewatte N, Paudyal P, Blyth FM, Coggon D, Crombez G, et al (2009) Evaluation of work-related psychosocial factors and regional musculoskeletal pain: results from a EULAR Task Force. Annals of the rheumatic diseases 68: 885–891. 10.1136/ard.2008.090829 18723563

[44] Bugajska J, Żołnierczyk-Zreda D, Jędryka-Góral A, Gasik R, Hildt-Ciupińska K, Malińska M, et al (2013) Psychological factors at work and musculoskeletal disorders: a one year prospective study. Rheumatology International 33: 2975–2983. 10.1007/s00296-013-2843-8 23934521 PMC3832752

[45] Cardoso JP, Ribeiro IdQB, Araújo TMd, Carvalho FM, Reis EJFBd (2009) Prevalence of musculoskeletal pain among teachers. Revista Brasileira de Epidemiologia 12: 604–614.

[46] Barros AJ, Hirakata VN (2003) Alternatives for logistic regression in cross-sectional studies: an empirical comparison of models that directly estimate the prevalence ratio. BMC Med Res Methodol 3: 21 10.1186/1471-2288-3-21 14567763 PMC521200

